# Extract mixture of plants (OXYLIA) inhibits fat accumulation by blocking FAS-related factors and promoting lipolysis via cAMP-dependent PKA activation

**DOI:** 10.29219/fnr.v68.10180

**Published:** 2024-03-12

**Authors:** Seong-Hoo Park, Sun-jung Baek, Minhee Lee, Hyun-A Shin, Hye jin Lee, Ok-Kyung Kim, Jeongmin Lee

**Affiliations:** 1 Department of Medical Nutrition, Kyung Hee University, Yongin, Republic of Korea; 2 ACROM CO., LTD. Suwon, Republic of Korea; 3 Division of Food and Nutrition and Human Ecology Research Institute, Chonnam National University, Gwangju, Republic of Korea

**Keywords:** obesity, lipolysis, lipogenesis, olive, rosemary, black bean

## Abstract

**Background:**

Obesity is characterized by an imbalance between energy intake and expenditure, leading to the excessive accumulation of triglycerides in adipose tissue.

**Objective:**

This study investigated the potential of Oxylia to prevent obesity in mice fed with a high-fat diet (HFD).

**Design:**

C57BL/6J mice were fed with one of the following five diets – AIN93G normal diet (normal control), 60% (HFD; control), HFD containing metformin at 40 mg/kg body weight (b.w.) (Met; positive control), HFD containing Oxylia at 30 mg/kg b.w. (O30), or HFD containing Oxylia at 60 mg/kg b.w. (O60) – for 15 weeks.

**Results:**

Mice under an HFD supplemented with Oxylia had decreased body weight gain, adipose tissue weight, and adipose tissue mass. In addition, triglyceride (TG), total cholesterol, and VLDL/LDL cholesterol levels were lower in the O60 groups than in the HFD-fed control group. Moreover, Oxylia supplementation decreased the expression of adipogenesis-related mRNAs and lipogenesis-related proteins while increasing the expression of lipolysis-related proteins in white adipose tissue and thermogenesis-related proteins in brown adipose tissue.

**Conclusions:**

These findings suggest that Oxylia has potential as a functional food ingredient for the prevention and treatment of obesity and related metabolic disorders.

## Popular scientific summary

- Supplementation with Oxylia conferred benefits on body weight, lipid profiles, and adipose mass in mice fed a high-fat diet.
- Oxylia supplementation regulated lipid metabolism, including adipogenesis, lipogenesis, and lipolysis in white adipose tissue, as well as thermogenesis in brown adipose tissue.

Obesity is a complex disease that arises from a combination of genetic, environmental, and lifestyle factors. Biochemically, obesity is characterized by an excess accumulation of adipose tissue, which results from an imbalance between energy intake and expenditure. Adipogenesis, de novo lipogenesis, and lipolysis are key biochemical processes involved in the development and regulation of adipose tissue, which is a highly dynamic endocrine organ that plays a central role in regulating energy homeostasis and metabolic health (1–3).

Adipogenesis, the process by which undifferentiated precursor cells develop into mature adipocytes, is a key factor in the development of obesity. Adipocyte differentiation is regulated by a complex network of transcription factors, including peroxisome proliferator activated receptor gamma (PPARγ), CAAT/enhancer-binding proteins (C/EBPs), and sterol regulatory element-binding protein 1 (SREBP-1) (4). On the other hand, lipogenesis is the process by which excess energy is converted into fatty acids and stored in adipose tissue. The regulation of lipogenesis involves a complex network of enzymes and transcription factors that are influenced by various hormonal and nutritional signals (5). Dysregulation of adipogenesis and lipogenesis can lead to an increase in the number and size of adipocytes, resulting in the expansion of adipose tissue and contributing to obesity (4, 5). As obesity progresses, the activation of lipase and β-oxidation of fatty acids in adipose tissue is suppressed, thereby inhibiting triglyceride (TG) breakdown, which may hasten the onset of obesity. The accumulation of excess TGs in adipose tissue can lead to inflammation and the release of adipokines, which can contribute to insulin resistance and the development of metabolic disorders such as type 2 diabetes (6, 7). The uncoupling protein (UCP)-mediated thermogenesis pathway plays a crucial role in TG breakdown by leading to energy expenditure in the form of heat. Therefore, stimulating lipolysis and thermogenesis is useful for preventing obesity and maintaining energy homeostasis (8).

Orlistat, phentermine-topiramate, and contrive are among the most commonly used obesity treatments; however, these drugs are associated with side effects such as diarrhea, oily stools, flatulence, cognitive as well as neuropsychiatric issues. Therefore, current research is focusing on the use of natural extracts for anti-obesity (9, 10).

Olive extract contains compounds such as oleuropein and hydroxytyrosol, which have demonstrated anti-obesity properties (11). Rosemary extract is rich in polyphenols, including rosmarinic acid and carnosic acid, which have been investigated for their potential anti-obesity effects. These compounds can modulate lipid metabolism by reducing lipid absorption and increasing fatty acid oxidation (12). Also, kidney bean extract, often containing phaseolamin, is known to inhibit the activity of alpha-amylase, an enzyme responsible for breaking down carbohydrates. By reducing carbohydrate digestion, kidney bean extract can potentially limit the post-meal increase in blood glucose levels and help in weight management (13). In addition, Micol et al. (14) suggested that a dietary supplement of Oxylia, formulated with a combination of olive, rosemary, and kidney bean extracts, can be taken as a safe adjuvant product for the weight loss management. They showed that the dietary supplement of Oxylia induced weight loss and a decrease in waist circumference during the 90 days of the treatment in a double-blind and placebo-controlled study. However, there is no evidence that the supplement changes metabolic function and weight loss-related molecular mechanisms. Thus, in the present study, we determined the effects of standardized Oxylia supplementation on adipogenesis, lipogenesis, and lipolysis in HFD-induced obese mice to elucidate the mechanism of the supplement’s anti-obesity effect.

## Materials and methods

### Preparation of a standardized sample of Oxylia

Oxylia, which contains olive (Olea europaea) leaf extract powder, rosemary (Rosmarinus officinalis L.) leaf extract powder, and black kidney bean (Phaseolus vulgaris L.) extract powder at a ratio of 6:3:1, was provided by Innovation Labo Technologies SL (Elche, Spain). Oxylia (0.5 g) was extracted with 50 mL of 100% methanol, and samples were filtered through a 0.45 μm nylon filter. Subsequently, 20 μL of the filtered sample was injected into the Agilent 1260 system equipped with a quaternary pump, autosampler, and diode array detector and with the column temperature set at 30°C. The column used was a Kromacil 100-5-C18 (250 mm × 4.6 mm id and 5 μm particle size), and standard products were purchased from Sigma Chemicals (St. Louis, MO, USA). The mobile phase consisted of 0.5% formic acid in H_2_O (A) and 0.5% formic acid in acetonitrile (B) (HPLC gradient of 10, 26, 65, 10, and 10% of solvent B at 0, 40, 60, 61, and 70 min, respectively, at a flow rate of 0.7 mL/min). The main index component was detected at 270 nm.

### Animals

Four-week-old male C57BL/6J mice from Saeron Bio (Uiwang, Korea) were housed under controlled conditions for 1 week prior to starting the experiment. For 15 weeks, the mice were fed with one of the five following diets: AIN93G normal diet (Normal diet, NC, normal control), 60% high-fat diet (HFD, C, obesity-induced control), HFD containing metformin at 40 mg/kg b.w. (Met, positive control), HFD containing Oxylia at 30 mg/kg b.w. (O30), or HFD containing Oxylia at 60 mg/kg b.w. (O60). This study was conducted in accordance with the guidelines and approved by the University Animal Care and Use Committee (KHGASP-21-562). Tissues and blood samples were collected from the mice after sacrifice.

### Biochemical analysis

The levels of TGs, total cholesterol, low-density lipoproteins (LDL)/high density lipoprotein (HDL) cholesterol, aspartate aminotransferase (AST), and alanine aminotransferase (ALT) were determined in the serum or feces using the instructions provided by the manufacturers of the respective assay kits. The kits used were the Triglyceride Quantification Kit (Biomax, Seoul, Korea), Cholesterol Assay Kit (Biomax), Aspartate Aminotransferase Activity Colorimetric Assay Kit (Biovision, Waltham, MA, USA), and Alanine Aminotransferase Activity Colorimetric Assay Kit (Biovision).

### Micro-CT

Mice were examined via abdominal tomography using micro-CT equipment (VIVA CT 80, Scano Medical AG, Switzerland).

### Quantitative real-time PCR

The RNeasy lysis (RLT) buffer (lysis buffer, Qiagen, Valencia, CA, USA) with β-mercaptoethanol was used to isolate total RNA from adipose tissue lysates. Subsequently, reverse-transcript II reverse transcriptase (Invitrogen, Carlsbad, CA) was used to generate cDNA from 1 μg of total RNA. Real-time polymerase chain reaction (PCR) was then conducted using Universal SYBR Green PCR Master Mix in accordance with the manufacturer’s instructions (Bio-Rad, CA, USA), and amplification was performed with selective primer sets detailed as follows: SREBP-1c (F: 5’-CCA GAG GGT GAG CCT GAC AA-3’, R: 5’-AGC CTC TGC AAT TTC CAG ATC T-3’), PPAR-γ (F: 5’-GCC CAC CAA CTT CGG AAT C-3’, R: 5’-TGC GAG TGG TCT TCC ATC AC-3’), C/EBPα (F: 5’-GAG CTG AGT GAG GCT CTC ATT CT-3’, R: 5’-TGG GAG GCA GAC GAA AAA AC-3’), and GAPDH (F: 5’-CAT GGC CTT CCG TGT TCC TA-3’, R: 5’-GCG GCA CGT CAG ATC CA-3’).

### Western blot

Mouse adipose tissues were lysed using a lysis reagent (Sigma, St. Louis, MO, USA), and an equal amount of protein (100 μg/lane) was separated using a 10% MiniPROTEAN® TGX™ Precast Protein Gel (Bio-Rad Laboratories). The separated proteins were transferred electrophoretically onto membranes using the Trans-Blot® Turbo^TM^ Transfer system (Bio-Rad). The membranes were then blocked, incubated with primary antibodies against p-AMP-activated protein kinase (AMPK), phosphorylated acetyl-CoA carboxylase (p-ACC), fatty acid synthase (FAS), leptin, adiponectin, lipoprotein lipase (LPL), protein kinase A (PKA), p-hormone-sensitive lipase (p-HSL), perilipin, adipose triglyceride lipase (ATGL), and β-actin (Cell Signaling, Beverly, MA, USA), and subsequently incubated with a secondary antibody (anti-rabbit IgG HRP-linked antibody, 1:5,000, Cell Signaling). The resulting protein bands were detected, quantified using CS Analyzer 3.0 (ATTO), and normalized using β-actin as a loading control.

### Measurement of intracellular cAMP concentration

A commercial enzyme immunoassay kit (Amersham Pharmacia Biotech, Piscataway, NJ, USA) was used to measure intracellular cAMP concentrations in adipose tissues, following the manufacturer’s instructions.

### Statistical analysis

The mean and standard deviation (SD) were used to express all data. SPSS PASW Statistic v.23.0 software (SPSS Inc., Chicago, IL, USA) was used to perform Analysis of variance (ANOVA) followed by a Duncan’s multiple range test for multiple comparisons. Statistical significance was determined at *P* < 0.05.

## Results

### Standardized Oxylia

**Fig. 1:**
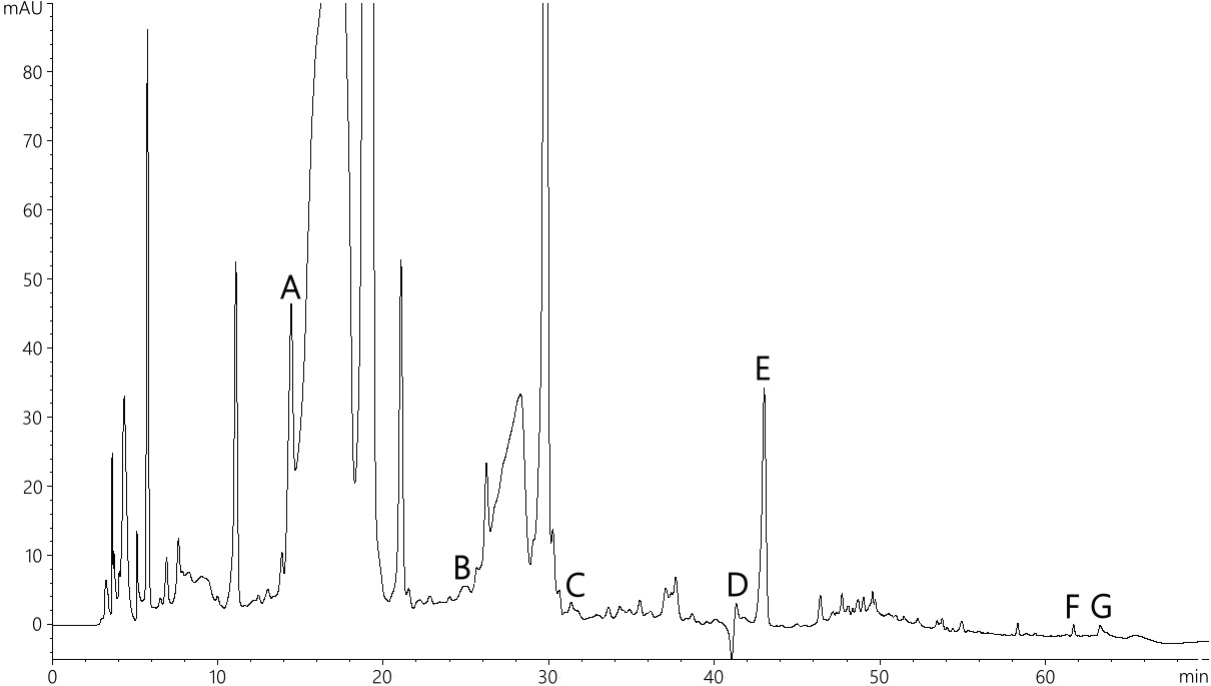
HPLC chromatograms at 270 nm of polyphenols in Oxylia. Peak identification: A, petunidin 3-glucoside; B, petunidin; C, malvidin; D, rosmarinic acid; E, oleuropein; F, carnosol; G, carnosic acid.

We found that Oxylia contained petunidin 3-glucoside, petunidin, and malvidin, which are anthocyanins of black kidney beans extracts; rosmarinic acid, carnosol, and carnosic acid, which are major compounds of rosemary extracts; and oleuropein, a major phenolic compound of olive extracts (Fig. 1).

### Effects of Oxylia on the body and organ weights of HFD-fed mice

We investigated the anti-obesity effect of dietary supplementation with Oxylia in HFD-fed obese rats by examining changes in body and organ weights. HFD-fed mice (Control) experienced marked increases in body weight, body weight gain, and food efficiency rate (FER) compared to the mice fed the normal control diet (NC). Compared to the HFD diet, a diet containing Oxylia was associated with a significant decrease in body weight and body weight gain. However, there was no difference in FER among Oxylia-fed groups, even compared to that of the HFD-fed control group, indicating that dietary supplementation with Oxylia did not influence experimental processes and results. There were no significant differences in kidney and spleen weight among the groups, but liver weight in the HFD-fed control group was 37.6% greater than that in the NC. In addition, the liver weight in mice supplemented with Oxylia and metformin as a positive control (Met) was significantly lower than that of the HFD-fed control group. Total adipose tissue weight was measured in three compartments: subcutaneous adipose tissue, visceral adipose tissue, which includes retroperitoneal and epididymal adipose tissue, and brown adipose tissue (BAT). Total, subcutaneous, and visceral white adipose tissue (WAT) weight significantly decreased with supplementation of Oxylia in a dose-dependent manner (*P* < 0.05). BAT weight also significantly decreased with supplementation of Oxylia, but there was no dose-dependent effect (Table 1).

**Table 1:** Effects of Oxylia on body weight, weight gain, and FER in high-fat diet (HFD)-induced obese mice.

Groups	NC	HFD supplementation			
		C	Met	O30	O60
**Initial body weight (g)**	20.99 ± 0.55^NS*^	21.88 ± 0.52	21.81 ± 0.61	21.31 ± 0.81	21.06 ± 0.71
**Final body weight (g)**	29.81 ± 1.17^e^	43.96 ± 1.19^a^	36.44 ± 1.45^d^	41.21 ± 2.88^b^	38.84 ± 1.35^c^
Weight gain**	8.82 ± 1.04^e^	22.08 ± 1.15^a^	14.63 ± 1.19^d^	19.90 ± 2.44^b^	17.78 ± 1.97^c^
FER***	5.12 ± 0.6^b^	7.72 ± 0.40^a^	7.07 ± 0.58^a^	6.96 ± 0.85^a^	7.74 ± 0.86^a^
**Organ weight (g)**					
** Liver**	1.21 ± 0.09^d^	1.94 ± 0.24^a^	1.42 ± 0.08^c^	1.65 ± 0.14^b^	1.59 ± 0.09^b^
** Kidney**	0.32 ± 0.02^NS*^	0.36 ± 0.04	0.35 ± 0.03	0.35 ± 0.03	0.36 ± 0.03
** Spleen**	0.09 ± 0.01^NS*^	0.10 ± 0.01	0.10 ± 0.01	0.10 ± 0.01	0.10 ± 0.01
**Adipose tissue weight (g)**					
** Total WAT**	1.88 ± 0.28^e^	6.09 ± 0.37^a^	3.88 ± 0.08^d^	5.25 ± 0.37^b^	4.33 ± 0.19^c^
** Subcutaneous WAT**	0.79 ± 0.14^e^	2.52 ± 0.21^a^	1.47 ± 0.18^d^	2.16 ± 0.22^db^	1.72 ± 0.09^c^
** Visceral WAT**	1.09 ± 3.57^d^	3.57 ± 0.28^a^	2.41 ± 0.13^c^	3.09 ± 0.29^b^	2.62 ± 0.18^c^
** BAT**	0.12 ± 0.02^d^	0.22 ± 0.03^a^	0.14 ± 0.01^c^	0.17 ± 0.01^b^	0.15 ± 0.02^bc^

NC, normal control; C, obesity-induced control; Met, metformin at 40 mg/kg b.w. (positive control); O30, Oxylia at 30 mg/kg b.w.; O60, Oxylia at 60 mg/kg b.w.; WAT, white adipose tissue. Values are presented as mean ± standard deviation (*n* = 8), and different superscripted letters indicate significance at *P* < 0.05. NS

* : No significant difference;

** Weight gain (g/15 weeks) = final body weight (g) − initial body weight (g);

*** FER (food efficiency rate) = weight gain (g)/total food consumption (g) × 100.

### Effects of Oxylia on lipid profiles in HFD-fed mice

We investigated the levels of lipid profiles, ALT, and AST in HFD-fed obese rats supplemented with Oxylia. Compared to the NC group, the HFD-fed control group showed significant increases in serum levels of TGs, total cholesterol, very low-density lipoproteins (VLDL)/LDL cholesterol, AST, and ALT. Metformin and Oxylia dietary supplementation resulted in significant decreases in these measures, except for HDL cholesterol, compared to the HFD-fed control group. Additionally, supplementation with Oxylia significantly increased the TGs and total cholesterol in feces (*P* < 0.05) (Table 2), indicating that Oxylia induces secretion by inhibition absorption of it.

**Table 2:** Effects of Oxylia on serum and feces lipid profiles and serum AST and ALT from high-fat diet (HFD)-induced obese mice.

Groups	NC	HFD supplementation			
		HFD	Met	O30	O60
**Serum**					
Total cholesterol (μg/μL)	13.24 ± 2.65^e^	37.76 ± 2.88^a^	21.74 ± 2.42^d^	29.09 ± 2.42^b^	25.03 ± 2.96^c^
Triglycerides (μg/μL)	11.35 ± 3.29^e^	30.22 ± 4.19^a^	17.96 ± 1.60^d^	25.00 ± 2.49^b^	21.72 ± 1.17^c^
VLDL/LDL cholesterol (μg/μL)	5.70 ± 1.26^c^	13.55 ± 1.36^a^	10.66 ± 0.91^b^	12.93 ± 1.39^a^	11.12 ± 1.70^b^
HDL cholesterol (μg/μL)	41.91 ± 5.66^b^	49.31 ± 5.09^a^	53.52 ± 6.95^a^	48.30 ± 3.67^a^	52.38 ± 4.75^a^
AST (mU/mL)	68.83 ± 4.12^b^	93.17 ± 14.31^a^	79.32 ± 14.40^ab^	83.80 ± 5.25^ab^	82.41 ± 10.86^ab^
ALT (mU/mL)	26.01 ± 5.49^b^	36.36 ± 5.18^a^	27.49 ± 3.24^b^	29.65 ± 3.89^b^	28.57 ± 5.59^b^
**Feces**					
Total cholesterol (μg/μL)	4.15 ± 0.61^d^	6.59 ± 0.59^c^	7.84 ± 0.68^b^	8.52 ± 0.48^b^	9.49 ± 0.71^a^
Triglycerides (μg/μL)	0.23 ± 0.05^e^	0.36 ± 0.04^d^	0.48 ± 0.04^c^	0.55 ± 0.06^b^	0.64 ± 0.06^a^

NC, normal control; C, obesity-induced control; Met, metformin at 40 mg/kg b.w. (positive control); O30, Oxylia at 30 mg/kg b.w.; O60, Oxylia at 60 mg/kg b.w. Values are presented as mean ± standard deviation (*n* = 8), and different superscripted letters indicate significance at *P* < 0.05.

### Effects of Oxylia on the adipose mass in HFD-fed mice

**Fig. 2:**
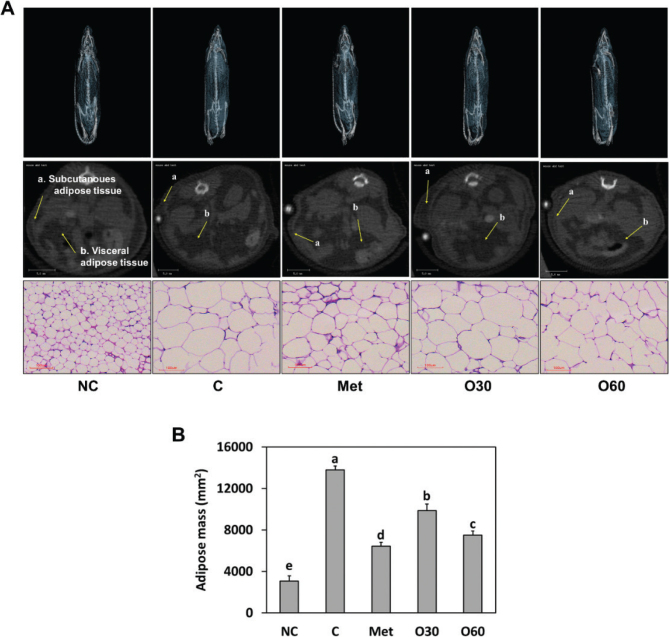
Effects of Oxylia on adipose tissue mass (A, microCT and H&E staining image; B, adipose mass) in high-fat diet (HFD)-induced obese mice. NC, normal control; C, obesity-induced control; Met, metformin at 40 mg/kg b.w. (positive control); O30, Oxylia at 30 mg/kg b.w.; O60, Oxylia at 60 mg/kg b.w. Values are presented as mean ± standard deviation (*n* = 8), and different superscripted letters indicate significance at *P* < 0.05.

We investigated the change of adipose mass in HFD-fed obese rats supplemented with Oxylia. Tomography analysis using micro-CT showed that adipose tissue mass in the control group significantly increased compared to that in the NC group. However, dietary supplementation with Oxylia significantly and dose-dependently decreased adipose tis-sue mass compared to the control diet. Additionally, the size of lipid droplets in the metformin and Oxylia at 60 mg/kg b.w. groups was significantly lower than that in the HFD-fed mice control group (*P* < 0.05) (Fig. 2). These findings suggest that Oxylia may inhibit body weight gain by decreasing adipose tissue mass and the size of adipocytes. We next examined the impact of Oxylia on lipid metabolism pathways in adipose tissues, including adipogenesis, lipogenesis, lipolysis, and thermogenesis, to investigate the biochemical mechanisms of this supplement’s effect.

### Effects of Oxylia on the adipogenesis and lipogenesis pathways of HFD-fed mice

**Fig. 3:**
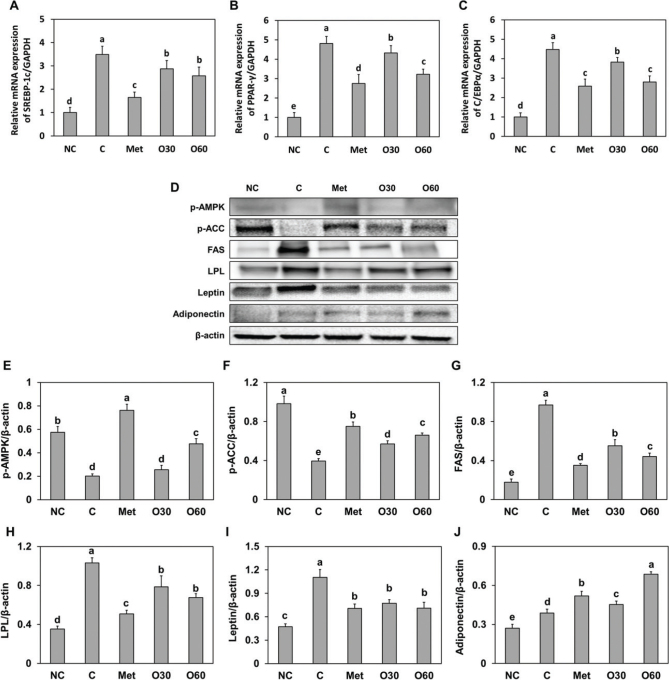
Effect of Oxylia on the mRNA expression of (A) SREBP1c, (B) PPAR-γ, and (C) C/EBPα and (D, band image) protein expression of (E) p-AMPK, (F) p-ACC, (G) FAS, (H) LPL, (I) leptin, and (J) adiponectin in white adipose tissue from high-fat diet (HFD)-induced obese mice. NC, normal control; C, obesity-induced control; Met, metformin at 40 mg/kg b.w. (positive control); O30, Oxylia at 30 mg/kg b.w.; O60, Oxylia at 60 mg/kg b.w. Values are presented as mean ± standard deviation (*n* = 8), and different superscripted letters indicate significance at *P* < 0.05.

We investigated the adipogenesis and lipogenesis pathways in HFD-fed obese rats supplemented with Oxylia. Compared to the HFD-fed control group, the groups supplemented with Oxylia showed significantly reduced messenger RNA (mRNA) expression of transcription factors such as SREBP1c, PPARγ, and C/EBPα (Fig. 3A–C). Meanwhile, the levels of de novo lipogenesis-related protein factors, such as phosphorylated AMPK and p-ACC, significantly increased in a dose-dependent manner in response to Oxylia supplementation compared to levels in the HFD-fed control group (*P* < 0.05). FAS and LPL were significantly lower in both the 30 and 60 mg/kg b.w. Oxylia supplementation groups than in the HFD-fed control group (Fig. 3D–H). In addition, compared to the HFD-fed control group, supplementation groups experienced a significant decrease in leptin protein expression and a significant increase in adiponectin protein expression (*P* < 0.05) (Fig. 3I and J). These findings suggest that Oxylia may regulate lipid metabolism by affecting the expression of lipogenic genes and transcription factors.

### Effects of Oxylia on the lipolysis and thermogenesis pathways of HFD-fed mice

**Fig. 4:**
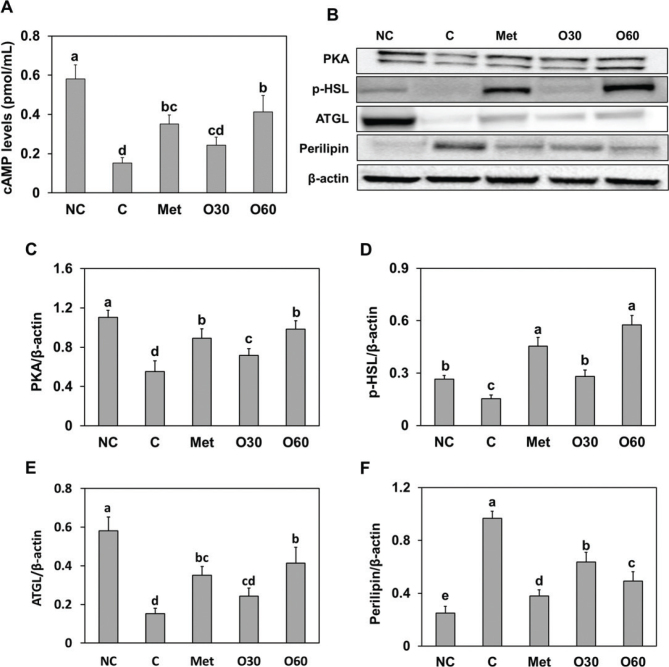
Effect of Oxylia on the (A) cAMP levels and (B, band image) protein expression of (C) PKA, (D) p-HSL, (E) ATGL, and (F) perilipin in white adipose tissue from high-fat diet (HFD)-induced obese mice. NC, normal control; C, obesity-induced control; Met, metformin at 40 mg/kg b.w. (positive control); O30, Oxylia at 30 mg/kg b.w.; O60, Oxylia at 60 mg/kg b.w. Values are presented as mean ± standard deviation (*n* = 8), and different superscripted letters indicate significance at *P* < 0.05.

We investigated the lipolysis and thermogenesis pathways in HFD-fed obese rats supplemented with Oxylia. Our findings showed that HFD supplementation led to the inhibition of proteins involved in the lipolysis pathway, specifically cAMP/PKA, HSL, ATGL, and perilipin. Conversely, Oxylia supplementation in HFD-fed mice significantly increased the expression levels of cAMP/PKA, p-HSL, and ATGL when compared to the HFD alone. Furthermore, Oxylia supplementation recovered perilipin protein expression, which was significantly increased in the control mice (*P* < 0.05) (Fig. 4).

**Fig. 5:**
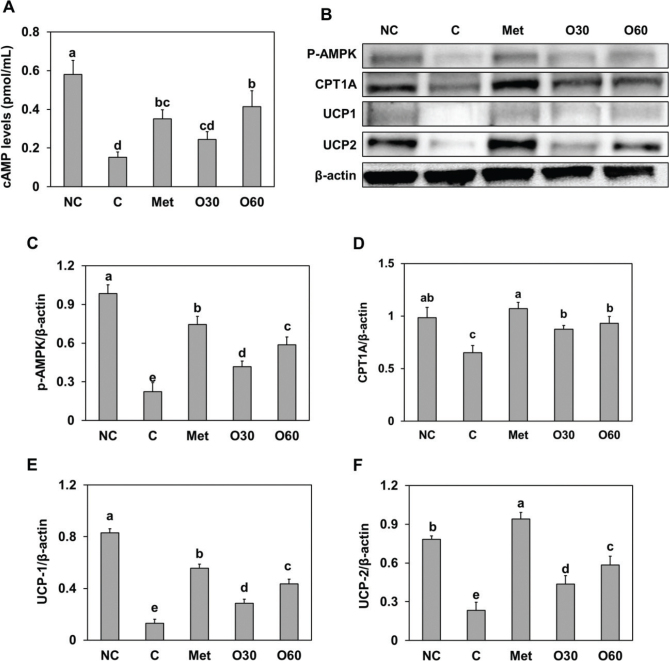
Effect of Oxylia on the (A) cAMP levels and (B, band image) protein expression of (C) p-AMPK, (D) CPT-1, (E) UCP-1, and (F) UCP-2 in brown adipose tissue from high-fat diet (HFD)-induced obese mice. NC, normal control; C, obesity-induced control; Met, metformin at 40 mg/kg b.w. (positive control); O30, Oxylia at 30 mg/kg b.w.; O60, Oxylia at 60 mg/kg b.w. Values are presented as mean ± standard deviation (*n* = 8), and different superscripted letters indicate significance at *P* < 0.05.

To investigate the effects of Oxylia on thermogenesis in BAT, we measured cAMP levels and expression levels of the p-AMPK proteins carnitine palmitoyltransferase 1 (CPT1), UCP-1, and UCP-2 in BAT. Compared to the HFD-fed control group, Oxylia-supplemented groups showed increased levels of cAMP expression and p-AMPK, CPT1, UCP1, and UCP-2 protein expression (*P* < 0.05) (Fig. 5). The upregulation of UCP1 and UCP2 protein expression in the Oxylia-supplemented groups suggests that Oxylia may have a beneficial effect on energy expenditure through the activation of the thermogenesis-related UCP pathway.

## Discussion

Obesity is an abnormal fat accumulation through complexly influenced by environment, diet, and low physical activity. The prevalence of the obesity and overweight was increased worldwide. The obesity is understanding the processes of adipogenesis, lipogenesis, and lipolysis. Adipogenesis refers to the formation and development of adipocytes (fat cells), while lipogenesis involves the synthesis of TGs within adipocytes, leading to fat accumulation. On the other hand, lipolysis is the breakdown of stored TGs into free fatty acids (FFAs) and glycerol (15–17).

We investigated the anti-obesity effect of dietary supplementation with Oxylia, a formulation containing olive, rosemary, and kidney bean extracts, in HFD-fed obese rats by examining changes in body and organ weights, lipid profiles, fat mass, and lipid metabolism. Previous research has shown that olive leaf extract has beneficial effects on obesity-related disorders such as dyslipidemia, insulin resistance, and inflammation (18–20). Hadrich et al. (18) used 3T3-L1 cells and HFD-fed rats to investigate the potential anti-insulin-resistance effects of oleuropein from olive leaf extract and found that the compound may contribute to weight loss and reduce obesity-related insulin resistance through several mechanisms. Similarly, Zhao et al. (21) showed that the supplementation of carnosic acid, which is found in rosemary extract, in the diets of HFD-induced obese mice significantly reduced body weight gain. In an evaluation of how anthocyanins extracted from black soybean affect body weight, adipose tissue weight, and serum lipids in rats fed with a HFD, Kwon et al. (22) found that black soybean anthocyanins lowered weight gain and improved lipid profiles. These findings support the potential of Oxylia, which also contains natural antioxidants, in managing obesity and obesity-related disorders. Therefore, in our present study, we investigated the effects of Oxylia on lipid metabolism in HFD-induced obese mice to elucidate the mechanism of the supplement’s anti-obesity effect. We found that HFD with Oxylia-fed mice groups were improved in body weight, liver weight, total subcutaneous and visceral WAT weight, and lipid profiles compared to HFD-fed control group.

The process of adipogenesis involves the activation of mitogen activated protein kinase (MAPK) and cAMP response element-binding protein (CREB), which then stimulate the transcription factors SREBP1, PPARγ, and C/EBPα in adipocytes, increasing lipid storage (23, 24). LPL activation induces the uptake of fatty acids from TGs of lipoproteins in the blood into adipocytes, where they are stored as TGs. The synthesis of new fatty acids through acetyl-CoA via the dephosphorylation of ACC and the activation of FAS plays a significant role in fat accumulation (24, 25). We found that that the expression levels of adipogenic transcription factors and lipogenesis-related lipogenic enzymes were decreased by HFD with Oxylia-fed mice groups compared to HFD-fed control group.

The lipolysis is the breakdown of stored TG into FFAs and glycerol. The HSL and ATGL enzymes are key players in lipolysis, and their regulation by hormones such as catecholamines, glucagon, and insulin is examined (26, 27). Also, excess fat accumulation is commonly attributed to an imbalance between energy intake and expenditure. The promotion of energy expenditure is particularly important for long-term clinical success in the treatment of obesity (28, 29). Fatty acids are generated from TG catabolism via the activation of lipase in adipocytes, which subsequently produces ATP via β-oxidation (30, 31). UCP1 is predominantly expressed in BAT and is responsible for non-shivering thermogenesis, which plays a critical role in energy balance and maintaining body temperature. UCP2, on the other hand, is expressed in a variety of tissues and is involved in regulating reactive oxygen species levels, as well as fatty acid metabolism (8, 32). We found that the expression levels of protein related to lipolysis and thermo-genesis pathways were improvement by HFD with Oxylia-fed mice groups compared to HFD-fed control group.

## Conclusions

The present study provides evidence that Oxylia supplementation may have beneficial effects on body weight, lipid profiles, adipose mass, and the regulation of lipid metabolism in HFD-fed mice. These findings suggest that Oxylia has potential as a functional food ingredient for the prevention and treatment of obesity and related metabolic disorders.

## Conflicts of interest and funding

The authors declare no conflict of interest. No funding was received.

## Author contributions

Conceptualization, S.-H.P., S.B., and J.L.; validation, S.-H.P., S.B., M.L., and O.-K.K.; formal analysis, S.-H.P. and S.B.; investigation, S.-H.P., S.B., and M.L.; resources, H.-A.S. and H.L.; data curation, S.-H.P., S.B., M.L., and O.-K.K.; writing –original draft preparation, M.L., O.-K.K., and J.L.; writing – review and editing, M.L., O.-K.K., and J.L. All authors have read and agreed to the published version of the manuscript.
